# Fixation matters: duration in fixative prior to immunofluorescent analysis directly impacts macrophage visualization in epithelial tissues

**DOI:** 10.1093/discim/kyag015

**Published:** 2026-07-23

**Authors:** Lizi M Hegarty, Erin Watson, Calum C Bain, Elaine Emmerson

**Affiliations:** Centre for Regenerative Medicine, Institute for Regeneration and Repair, University of Edinburgh, Edinburgh, UK; Centre for Regenerative Medicine, Institute for Regeneration and Repair, University of Edinburgh, Edinburgh, UK; School of Infection and Immunity, University of Glasgow, Glasgow, UK; Centre for Regenerative Medicine, Institute for Regeneration and Repair, University of Edinburgh, Edinburgh, UK

**Keywords:** macrophages, immunofluorescence, fixation, microscopy, epithelia

## Abstract

**Introduction:**

Macrophages are now recognized as key players in a range of tissues and biological processes, responding to injury and infection, and facilitating development and regeneration. As the importance of macrophage crosstalk within these processes has been revealed, so too has the significance of studying the spatial positioning of macrophages within the tissue of interest. As such, immunofluorescent microscopy-based analysis is becoming an increasingly attractive technique for immunology research. While tissue fixation preserves the tissue architecture and immobilizes target antigens, prolonged fixation can negatively impact protein recognition.

**Methods:**

We compared three different durations of tissue fixation and the expression of key macrophage and structural cell markers in the submandibular gland, pancreas, kidney, and skin by immunofluorescent imaging.

**Results:**

We report that prolonged exposure to a paraformaldehyde-based fixative profoundly impacts detection of cell surface markers that define macrophage subsets in the mouse submandibular gland, in contrast to epithelial cell markers, which appear more robust. We find that this is not exclusive to the salivary gland, and similar effects are seen in the pancreas and kidney. Importantly, a short duration of fixation allowed the detection of macrophage subsets in both mouse and human tissue without compromising the detection of other markers.

**Conclusion:**

Adoption of a short fixation approach enables accurate detection of a wide range of cell types in tissues, and facilitates exploration of spatial positioning and cell proximity by immunofluorescent microscopy analysis.

## Graphical Abstract

**Graphical Abstract:**
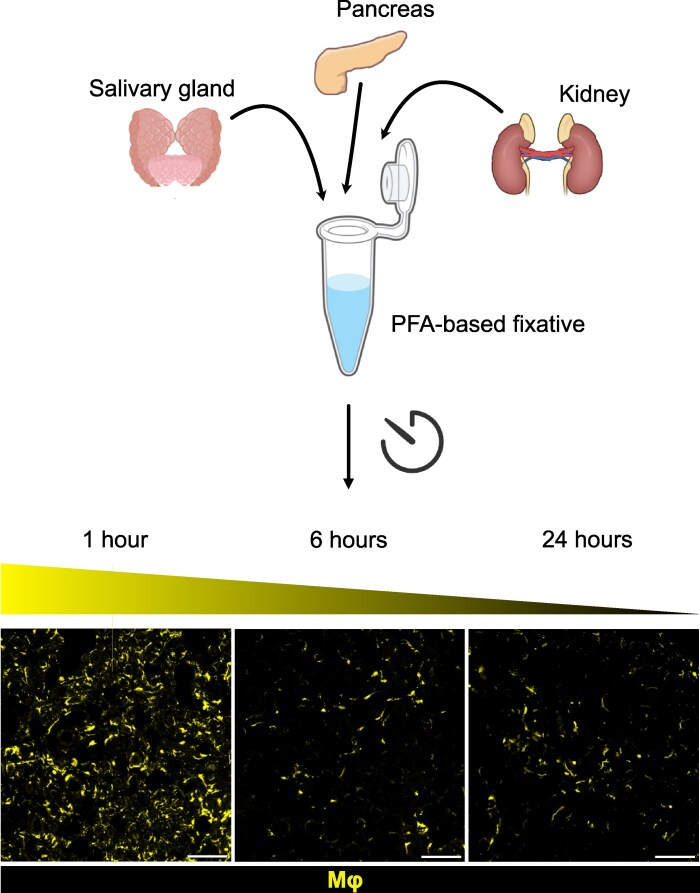

## Introduction

Macrophages are innate immune cells performing a spectrum of functions. These include immune surveillance and defence, encompassing specific processes such as phagocytosis, antigen presentation and cytokine secretion [1]. More recently, these functions have been expanded to include a vast range of roles beyond immunity, and macrophages are now also recognized as important regulators of homeostasis and repair, where they contribute by secreting growth factors and mediating metabolism [2].

Immunology research has traditionally relied on flow cytometry [3] to ascertain the heterogeneity of immune cells within tissues in health, following infection and injury, or during inflammatory resolution or tissue regeneration. However, it is becoming increasingly evident that cellular positioning in the tissue is important [4–6], and this spatial data is lost when the tissue is dissociated prior to analysis, such as with flow cytometry. Furthermore, many cells which provide a niche for immune cells in the tissue, such as neurons, do not survive traditional tissue digest and/or are poorly captured by flow cytometry analysis. Thus, many researchers now combine flow cytometry and immunofluorescent microscopy-based analysis for a thorough characterization of the immune environment.

Immunofluorescent staining has become a fundamental method to detect and visualize a multitude of different cell types *in situ*. The technique involves incubation of the tissue with a primary antibody which recognizes and binds to a specific antigen or epitope, followed by detection by a fluorescently conjugated secondary antibody, which recognizes the host species of the primary antibody. Alternatively, directly conjugated antibodies provide a one-step staining method. Tissue fixation is an essential step in tissue histology and immunofluorescent staining alike, and acts to not only prevent the tissue from degrading but also to immobilize target antigens and preserve the tissue architecture [7]. Furthermore, it is an important step to make tissues safe to work with by inactivating viruses and bacteria. However, excessive fixation can negatively impact protein recognition [8], ultimately impacting data interpretation.

We have previously shown that the murine submandibular gland (SMG) harbours at least two distinct macrophage subsets: a CD11c^+^CD163^−^ population and a CD11c^−^CD163^+^ population [9]. Crucially, we find that these subsets are colocalized with different cell types in the SMG: with CD11c^+^CD163^−^ cells more frequently associated with epithelial cells, while CD11c^−^CD163^+^ cells appear to associate more with structures such as blood vessels [9]. The positioning of CD11c^−^CD163^+^ cells in the SMG is in agreement with so-called ‘TLF’ (TIM4 and/or LYVE-1 and/or FRβ–expressing) macrophages, which have been reported across multiple different tissues [10–12].

Here, we report that the duration of tissue fixation can profoundly impact the extent of detection of cell surface markers that define macrophage subsets in the mouse SMG. This contrasts with corresponding surface markers on other cell lineages, such as epithelial cell markers, which appear more robust and unaffected by the same fixation process. Furthermore, by a comparative analysis of fixation protocols across a number of tissues, we show that this is not exclusive to the SMG, and similar effects are seen in a number of other organs. Thus, it is crucial to ensure that fixation protocols are carefully applied to ensure they are appropriate for the tissue and cell type of interest.

## Materials and methods

### Animal experiments

All animal experiments adhere to the NC3Rs ARRIVE guidelines and the University of Edinburgh guidelines on the care and use of laboratory animals. All tissues were collected following an approved UK Home Office euthanasia method. Several studies have highlighted that sexual dimorphism exists in mouse SMG structure, transcriptome and immune cells [9, 13–15], skin immunity [16], and pancreatic [17] and kidney [18] gene expression; thus, for simplicity and in order to remove any confounding factors, only male mice were used in this study. Mice were aged between 8 and 12 weeks.

### Human tissue

Human submandibular salivary gland was collected from patients undergoing tissue resection, and from tissue which would otherwise be discarded, under the NHS Lothian BioResource (approval numbers SR857 and SR2337) and with patient consent. Further details are included in Table 1.

**Table 1.** Additional details about human tissue used in this study.

Sample ID	Sex	Age	Fixation
EMM-H-A-001	Male	81	24 hours in 4% PFA
EMM-H-A-003	Male	69	24 hours in 4% PFA
SR857-1	Female	72	6 hours in 4% PFA
SR857-4	Male	58	6 hours in 4% PFA
SR857-5	Female	68	1 hour in Antigen Fix
SR2337-1	Female	42	1 hour in Antigen Fix

### Tissue processing

SMGs, kidneys, pancreas, and dorsal skin were fixed for either 1, 6 or 24 hours in Antigen Fix (Diapath), a paraformaldehyde-based fixative, at 4°C, followed by 3× washes with phosphate-buffered saline (PBS; Merck). After fixation, tissue was incubated in 34% sucrose (Sigma-Aldrich) overnight at 4°C before embedding in OCT (Leica). Twenty-micrometre sections were cut using a cryostat (Leica) and stored at −20°C.

### Immunofluorescent analysis

For each marker of interest, all samples were stained at the same time for consistency. Slides were allowed to come to room temperature. Tissue sections were permeabilized with ice-cold acetone/methanol (1:1) for 1 min. Sections were then air-dried for 2 minutes, followed by washing in Wash Buffer (PBS + 0.2% BSA) for 3 minutes. Tissue was blocked for 20 minutes at room temperature with Blocking Buffer (1:500 FC Block [Biolegend # 101320] in PBS + 1% BSA). Sections were incubated with primary antibodies overnight at room temperature. Antibodies are listed in Table 2. Antibodies were detected using donkey AF488-, Cy3-, or AF647-conjugated secondary Fab fragment antibodies (Jackson Laboratories) and nuclei stained using Hoechst 33342 (1:1000, Sigma-Aldrich), and mounted using Prolong Gold anti-fade mounting media. Images of three random fields of view per sample were acquired on a Leica SP8 confocal microscope. For each marker of interest, microscope settings and laser power were kept consistent across samples. Laser parameters are listed in Table 3. Fluorescent images were collated with NIH ImageJ software and quantified using QuPath software (macrophage cell counts) or ImageJ (epithelial/endothelial intensity).

**Table 2.** Primary antibodies used for immunofluorescent staining.

Antibody	Clone	Species	Supplier	Cat #	Dilution	RRID
AQP5	EPR3747	Rabbit	Abcam	ab92320	1:200	AB_2049171
CD11c	N418	Armenian Hamster	Biolegend	117302	1:100	AB_313770
CD163	TNKUPJ	Rat	Thermo Fisher	14-1631-82	1:500	AB_2716934
CD31		Goat	R&D Systems	AF3628	1:200	AB_2161028
ECAD	ECCD-2	Rat	Invitrogen	13-1900	1:400	AB_2533005
IBA1	EPR16588	Rabbit	Abcam	ab178846	1:500	AB_2636859

**Table 3.** Laser parameters used for immunofluorescent imaging.

Marker	Fluorophore	Laser power (%)	Laser gain	Detector
AQP5	AF-488	2	10	HyD Detector
CD11c	AF-555	2	600	PMT Detector
CD163	AF-488	0.7	10	HyD Detector
CD31	AF-647	2	20	HyD Detector
ECAD	Cy3	1	450	PMT Detector
IBA1	AF-647	1.3	20	HyD Detector
Hoechst	UV	1.2	600	PMT Detector

## Results

We have recently demonstrated that macrophages are the predominant immune cell type in the murine submandibular gland (SMG) and that they can be separated into at least two distinct populations: a CD11c^+^CD163^−^ population and a CD11c^−^CD163^+^ population [9]. However, our early analysis of tissue fixed for 6 hours found that the latter population was minor in comparison to their CD11c^+^CD163^−^ counterparts when assessed by immunofluorescent staining. This was corroborated when we analysed these populations by methods that require tissue dissociation, such as flow cytometry and single-cell RNA sequencing (scRNAseq) [9]. However, this is at odds with findings in other mucosal tissues such as the intestine [19, 20] and skin [21], where these different macrophage subsets are found in relatively similar abundance. Importantly, there is evidence that CD163^+^ macrophages inhabit different anatomical niches within tissues when compared to their CD163^−^ counterparts [10], and as such, being able to accurately locate these cells *in situ* in tissue is becoming increasingly important.

**Figure 1:**
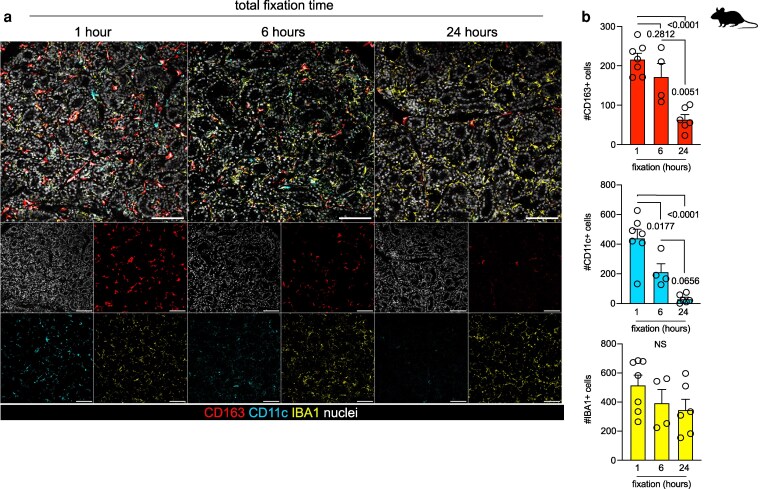
Prolonged tissue fixation impacts macrophage visualization in the mouse submandibular gland. (a) Representative expression of CD163, CD11c and IBA1 in SMG tissue from adult male C57BL/6J mice, fixed for 1, 6 or 24 hours. Large images show composites of all channels; insets display individual channels. Scale bar = 100 μm. (b) Graphical enumeration of CD163^+^, CD11c^+^ and IBA1^+^ cells in the mouse SMG at the indicated time points post-fixation. Data were obtained from three fields of view from 4 to 7 mice per timepoint. Statistical testing was undertaken using one-way ANOVA with Tukey *Q* post-hoc testing. Abbreviation: NS, non-significant.

Here, we tested three different durations of tissue fixation and compared expression of some key macrophage and structural cell markers in the SMG. We analysed adult (8–12 weeks of age) male murine submandibular gland (SMG) which had been fixed in Antigen Fix solution (Diapath), a paraformaldehyde-based fixative agent. We compared SMG that had been fixed for 24 hours (i.e. overnight)—a common fixation protocol in salivary gland research [22–26]—with two shorter periods of 1 or 6 hours (as used in [27–29]). We first stained cryosections for the pan-macrophage marker IBA1, and for the subset markers CD11c and CD163. We found that there were no significant differences in the number of IBA^+^ macrophages between SMG that had been fixed for 1, 6 or 24 hours (Fig. 1a and b). Conversely, we found that CD163^+^ macrophages in the SMG were abundant after 1 hour of fixation, but were reduced with increased fixation time, significantly so after 24 hours (Fig. 1a and b). Similarly, we found that CD11c^+^ macrophages were reliably visible with 1 hour of fixation, and strikingly reduced with either 6 or 24 hours fixation (Fig. 1a and b). This indicates that while IBA1 staining is robust and detectable across a range of fixation times, expression of CD163 and CD11c in the SMG is notably negatively affected by prolonged fixation of the tissue.

**Figure 2:**
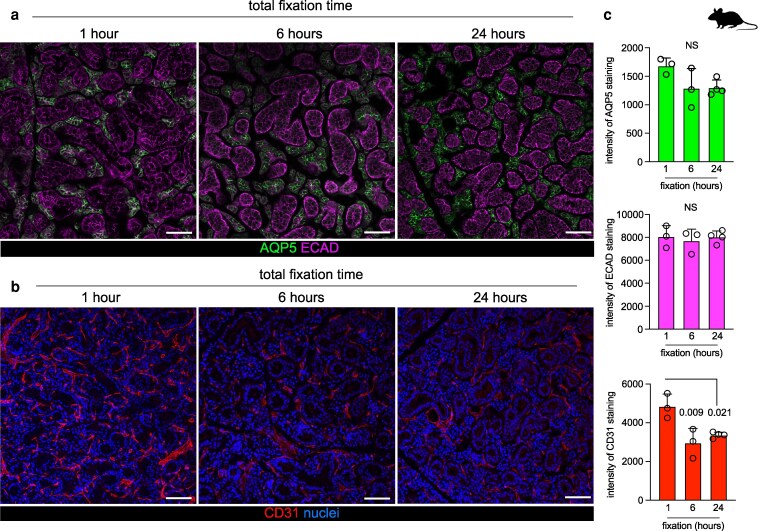
Prolonged fixation impacts submandibular gland endothelial markers but not epithelial markers. (a) Representative expression of aquaporin-5 (AQP5) and E-cadherin (ECAD) in SMG tissue from adult male C57BL/6J mice, fixed for 1, 6 or 24 hours. Scale bar = 100 μm. (b) Representative expression of CD31 (PECAM-1) in SMG tissue from adult male C57BL/6J mice, fixed for 1, 6, or 24 hours. Scale bar = 100 μm. (c) Graphical enumeration of AQP5, ECAD and CD31 intensity in the mouse SMG at the indicated time points post-fixation. Data were obtained from three fields of view from 3 to 4 mice per timepoint. Statistical testing was undertaken using one-way ANOVA with Tukey *Q* post-hoc testing. Abbreviation: NS, non-significant.

We then extended our analyses to investigate epithelial markers in the SMG. Epithelial integrity and/or expression of epithelial markers is routinely used in salivary gland research to assess tissue function and regeneration/degeneration following injury. In this study, we chose to analyse the pan-epithelial marker E-Cadherin because it is a crucial cell-to-cell adhesion molecule which is found in nearly all normal epithelial tissues, and thus it has relevance beyond just the salivary gland [30]. Macrophages are known to intimately associate with epithelial cells and blood vessels in other organs [10, 31–33]; and as such, being able to accurately visualize interactions is crucial. We found that E-cadherin (ECAD) and the acinar cell-specific water channel aquaporin-5 (AQP5) appeared comparable across all fixation conditions (Fig. 2a and c). This suggests that these epitopes are robust and can be reliably detected regardless of the fixation length. In contrast, while the endothelial cell marker CD31 (PECAM-1), which is often used to mark blood vessels, was apparent after 6 and 24 hours of fixation, the expression was notably stronger after only 1 hour of fixation (Fig. 2b and c). This implicates over-fixation as a potential cause for concern when assessing the vascularization of tissue or the nuances of endothelial barrier function.

**Figure 3:**
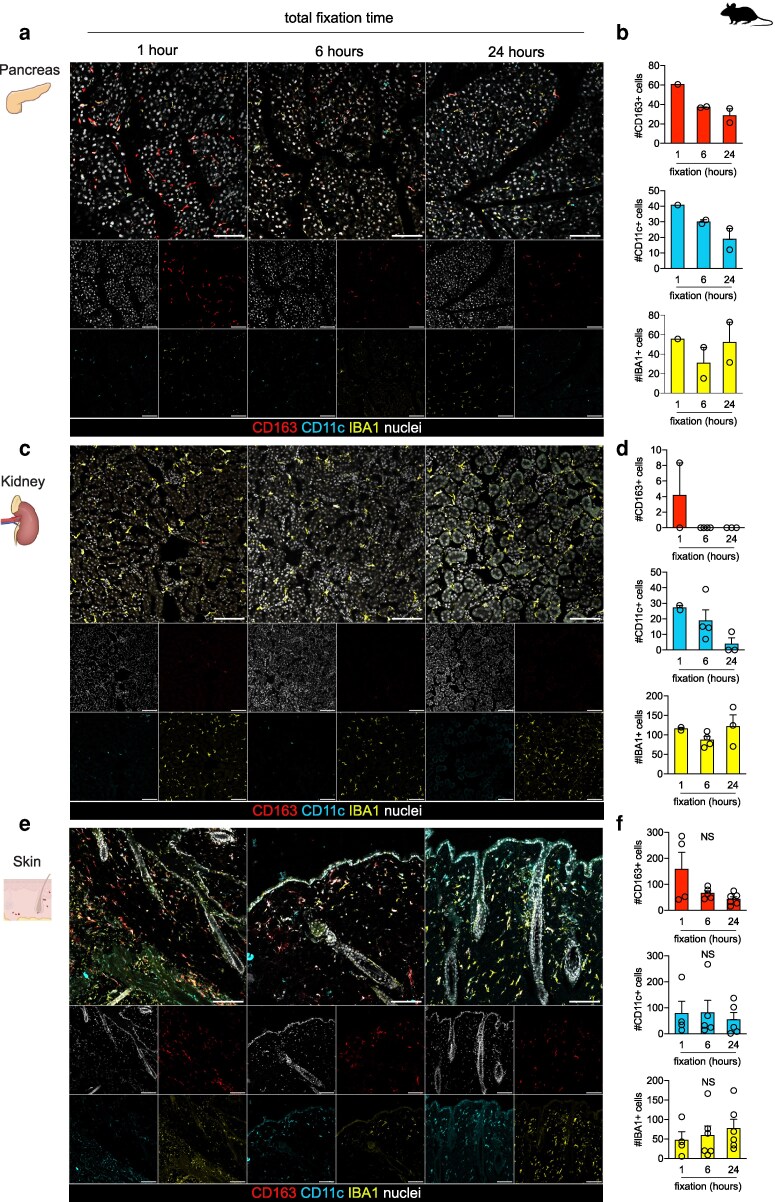
Sensitivity of macrophages to prolonged fixation is tissue-specific. Representative expression of CD163, CD11c, and IBA1 in (a) pancreas; (c) kidney; and (d) skin from adult male C57BL/6J mice, fixed for 1, 6 or 24 hours. Large images show composites of all channels; insets display individual channels. Scale bar = 100 μm. Graphical enumeration of CD163^+^ (b), CD11c^+^ (d) and IBA1^+^ (f) cells in the mouse SMG at the indicated time points post-fixation. Data were obtained from three fields of view from 1 to 6 mice per timepoint. Where appropriate, statistical testing was undertaken using one-way ANOVA with Tukey *Q* post-hoc testing. Abbreviation: NS, non-significant.

We next set out to find out if the duration of fixation also influenced the visualization of macrophage subsets in other epithelial tissues. We analyzed the mouse pancreas, an organ where roles for macrophages in development and regeneration have recently been described [34, 35]; the kidney, since this is an organ where an interest in macrophages is flourishing, albeit with limited utility of or success at visualizing macrophages by immunofluorescence [36–38]; and skin, an organ that has long been known to harbour macrophages with roles in repair and regeneration [39, 40]. Consistent with the SMG, we found that the pancreas, kidney, and skin all harbour IBA1^+^, CD11c^+^ and CD163^+^ macrophages (Fig. 3). Similar to the SMG, the detection of IBA1^+^ macrophages in the pancreas appeared unaffected with the increased duration of fixation, while expression of CD163 appeared to be reduced (Fig. 3a and b). Intriguingly, CD11c expression seemed lower in the pancreas than the SMG in all fixation conditions, while also appearing to become sparser with fixation duration (Fig. 3a and b). We also analysed mouse kidney and detected IBA1^+^, CD163^+^ and CD11c^+^ cells, albeit in small numbers for the latter two (Fig. 3c and d). However, the detection of CD163 and CD11c appeared reduced with longer fixation times (Fig. 3c and d). Finally, we analysed mouse dorsal skin fixed for 1, 6 or 24 hours and sectioned in cross-section. Unlike the other epithelial organs that we analysed, we found that CD11c^+^, CD163^+^ and IBA1^+^ cells were all abundant in the dermis after 1 hour of fixation, and their numbers remained consistent after 6- and 24 hours fixation (Fig. 3e and f). Collectively, this demonstrates that the duration of fixation negatively impacts visualization of macrophage subsets in other epithelial organs, in addition to the salivary glands, but that this effect is highly tissue-specific.

**Figure 4:**
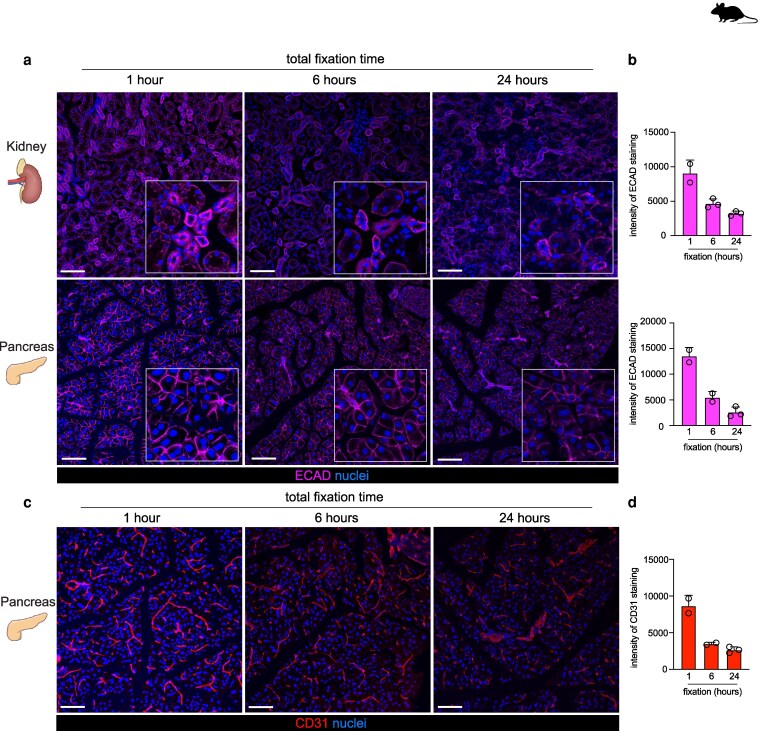
Structural markers are influenced by over-fixation in a tissue-specific manner. (a) Representative expression of ECAD in kidney and pancreas from adult male C57BL/6J mice, fixed for 1, 6 or 24 hours. Insets display magnified (×4) images. Scale bar = 100 μm. (b) Graphical enumeration of ECAD intensity in mouse kidney and pancreas at the indicated time points post-fixation. Data were obtained from three fields of view from 2 to 3 mice per timepoint. Due to the small sample sizes in some groups statistical testing could not be undertaken. (c) Representative expression of CD31 in pancreas from adult male C57BL/6J mice, fixed for 1, 6 or 24 hours. Scale bar = 100 μm. (d) Graphical enumeration of CD31 intensity in mouse pancreas at the indicated time points post-fixation. Data were obtained from three fields of view from 2 to 3 mice per timepoint. Due to the small sample sizes in some groups, statistical testing could not be undertaken.

In addition, we analysed the pancreas and kidney for the epithelial marker, ECAD, and the endothelial marker, CD31. While expression of ECAD in the pancreas was evident across fixation conditions, similar to the SMG, albeit with reduced intensity, fixation of the kidney for 6 and 24 hours resulted in clearly disrupted epithelial staining when compared to 1 hour (Fig. 4a and b). Similar to the SMG, CD31 staining was strongest after 1 hour of fixation and expression intensity appeared to decline at 6 and 24 hours (Fig. 4c and d). Intriguingly, CD31 expression was not visible in the kidney with any of the fixation conditions analysed (not shown). This could reflect that CD31^+^ is not a good marker for vasculature in the kidney, but may also highlight the sensitivity of the kidney to fixation when analysing by immunofluorescence.

**Figure 5:**
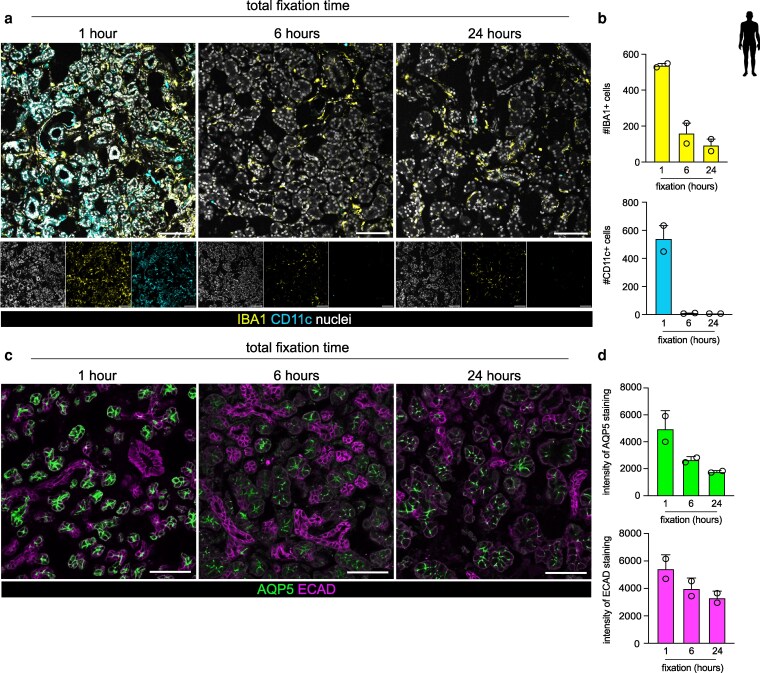
Duration of tissue fixation impacts macrophage visualization in the human submandibular gland. (a) Representative expression of CD11c and IBA1 in human submandibular gland (hSMG), fixed for 1 hour, 6 or 24 hours. Scale bar = 100 μm. (b) Graphical enumeration of CD11c^+^ and IBA1^+^ cells in human SMG at the indicated time points post-fixation. Data were obtained from three fields of view from two donors per timepoint. Due to the small sample sizes, statistical testing could not be undertaken. (c) Representative expression of aquaporin-5 (AQP5) and E-cadherin (ECAD) in human submandibular gland (hSMG), fixed for 1, 6, or 24 hours. Scale bar = 100 μm. (d) Graphical enumeration of AQP5 and ECAD intensity in human SMG at the indicated time points post-fixation. Data were obtained from three fields of view from two donors per timepoint. Due to the small sample sizes, statistical testing could not be undertaken.

Finally, we investigated whether we saw a similar sensitivity to fixation time in human salivary gland samples. We analysed archived human submandibular gland (hSMG) which had been fixed for 1 hour in Antigen Fix, or for either 6 or 24 hours with 4% PFA, and stained for IBA1 and CD11c (Fig. 5). We found clear CD11c and IBA1 staining in hSMG fixed for 1 hour, whereas like in mouse SMG, CD11c detection was reduced with 6 and 24 hours. Unlike mouse SMG, IBA1 also appeared to be sensitive to the longer durations of fixation in hSMG, with reduced staining from 6–24 hours of fixation (Fig. 5a and b). However, it is possible that this could be a reflection of the differences between 4% PFA and Antigen Fix. Finally, we found that the epithelial markers ECAD and AQP5 were evident under all fixation conditions (Fig. 5c and d). Importantly, 1 hour of fixation allowed the detection of macrophage subsets in both mouse and human SMG without compromising the detection of other markers, including IBA1, aquaporin-5, E-cadherin and CD31

Overall, our results demonstrate that the duration of fixation must be carefully considered on a tissue-specific basis while taking into account the target proteins of interest, in order to prevent loss of detection and inaccurate conclusions.

## Discussion

Macrophages are now known as key mediators of a multitude of cellular processes beyond their classical phagocytic role, including processes related to repair and regeneration, among others [2]. Indeed, we have previously shown that macrophages in the submandibular salivary gland (SMG) are crucial in the acute response to irradiation injury [9]. However, tissue-resident macrophages are not homogenous, and the heterogeneity reported in numerous tissues often relates to their ontogeny, longevity, function and positioning [41]. Importantly, their expression profile can often inform on their function and anatomical location. For example, border-associated macrophages and microglia in the brain are thought to be distinguishable by their expression, or lack thereof, of CD206, CD163 and CD169 [42], markers which are often associated with proximity to blood vessels and scavenging functions. In contrast, IBA1 is considered to be a more generic macrophage marker [43, 44]. We and others have previously shown that there are at least two transcriptionally distinct subsets of macrophages in the mouse SMG, inhabiting distinct anatomical niches, and which can be separated by their expression of CD11c and CD163 [9, 22]. These findings are in line with recent studies by the Epelman group, who report that similar macrophage subsets are found across 17 murine tissues [10]; while other studies have reported macrophage separation on the basis of CD11c and CD163 expression in colon [19, 20] and skin [21].

In this study, we explored whether the duration of tissue fixation impacted the ability to detect these subsets in the SMG by immunofluorescence. We found that while macrophages marked by either CD11c or CD163 were abundant after 1 hour of fixation, this significantly declined when the tissue was fixed for longer periods of 6 or 24 hours, suggesting a sensitivity with prolonged fixation. In contrast, detection of the pan-macrophage marker IBA1, or the epithelial markers aquaporin-5 and E-cadherin, did not appear to change with increased fixation. A number of published studies have aimed to explore aspects of macrophage biology in the mouse submandibular gland using histological techniques. However, many of these studies find a dearth of macrophages in homeostatic conditions when immunofluorescence is used [22, 45, 46]; a finding which is at odds with other studies which use a combination of methods to assess cellular composition of the tissue [9, 47, 48]. Thus, in order to ensure that we accurately analyse macrophage abundance and subsets in the future, we have subsequently amended our tissue fixation protocol for immunofluorescent analysis. We find that with a short (1 hour) fixation, we preserve both the epitopes and architecture of structural cells (such as epithelial markers) while also gaining a more representative view of macrophage abundance and subsets.

Crucially, we found that the sensitivity of these macrophage subsets was not restricted to the salivary gland; kidney, and pancreas also showed a similar propensity to lose the signal of CD11c^+^ and CD163^+^ cells with increased fixation, albeit with a small sample size, while skin was visibly more robust. In this study, we have not accounted for the fact that cells beyond macrophages, including dendritic cells, monocytes, and some T and NK cells, can also express CD11c [49]. However, by coupling with a pan-macrophage marker, such as IBA1, we are able to make such a distinction using immunofluorescence. While macrophages express different and varying levels of a plethora of markers across different tissues [50], our study highlights that fixation has a profound impact on the visualization of such markers in a range of tissues. Importantly, we find that while some important tissue structures, such as epithelia, are seemingly less affected by fixation, detection of other structures which provide a niche for tissue macrophages, and vice versa, including the blood vessels [51–53], is affected. This is in agreement with reports that paraformaldehyde (PFA) fixation can result in a loss of CD31 signal in brain tissue [54], and in line with a recently developed protocol for assessing CD31 in the mouse eye which instead advises fixation for as little as 50 minutes [55]. The reasons for the disparity in response to prolonged fixation that we observe may be associated with the type of protein being detected. For example, integrins, such as CD11c, are known to be highly sensitive to tissue processing and can become deformed, leading to changes in antibody binding [56]. Furthermore, PFA-based fixation can create crosslinks that obscure epitopes or interfere with antibody binding [54]. Similarly, scavenger receptors, such as CD163, are sensitive to tissue processing and prone to cleavage [57]. Conversely, proteins such as IBA1 are more stable and are intriguingly non-reactive with gentler fixation methods such as glyoxal [58]. Furthermore, PFA-based fixatives are highly effective at preserving cadherins, such as E-cadherin, and maintaining their spatial localization at adheren junctions [59], which may explain their relative robustness.

Both human and mouse SMGs exhibit a similar epithelial structure, and both contain a mix of serous and mucous acinar cells. The primary difference between the species is that mice exhibit sexual dimorphism in the structure of the SMG, and male mouse SMGs contain granular convoluted tubules, which produce growth factors and are not present in female mouse SMGs. In contrast, human SMGs do not exhibit sexual dimorphism [60]. Importantly, both mouse and human SMGs are susceptible to physical injury and insult resulting from exposure to external factors, such as radiation, infection, and excessive inflammation. Importantly, mice exhibit similar acute and degenerative changes to humans in response to such insults, making the mouse a common research model. In this study, we found that while mouse IBA1 was unaffected by the duration of fixation in mouse SMG, longer periods of fixation in human SMG resulted in fewer IBA1^+^ cells. However, the human SMG included in this study was analysed retrospectively, whereas mouse tissue was collected specifically to compare the duration of fixation. Mouse tissues were all fixed using Antigen Fix, whereas human SMG fixed for 6 and 24 hours, used 4% PFA, in line with our protocols at the time of collection. Thus, it is possible that 4% PFA has a more profound effect on IBA1 than Antigen Fix, even when incubated for the same amount of time. It is conceivable that Antigen Fix is more aligned to 2% PFA, a gentler fixative approach which has been used in recent immunology studies [61–63]. Moreover, human tissue inherently introduces many more variables when compared to mouse studies (sex, age, ethnicity, health status). We cannot rule out that such variables also contribute to the observed differences. Despite this, we remain confident that shortening the duration of tissue fixation allows for accurate detection of proteins of interest without compromising valuable data, in both mouse and human tissue.

Excessive cross-linking of proteins by prolonged exposure to formalin masks antigenic epitopes, and consequently can prevent antibodies from binding effectively. Overall, our data support studies which have found that increased fixation impacts the fluorescent signal when analysing oligodendrocyte precursor cells (OPCs), or complement staining in the brain, purportedly due to masking of the antigen [64–66]. To the best of our knowledge, this is the first report of differences in tissue macrophage abundance with increased fixation. Ultimately, our findings reveal that macrophage visualization is improved while tissue integrity is not compromised with short fixation; thus, providing an advantage over prolonged fixation. By adopting this tissue processing approach, users can accurately quantify a wide range of cell types in tissues, while also exploring their spatial positioning and proximity to each other.

## Data Availability

The data underlying this article are available in the article and in the accompanying supplementary material.

## Abbreviations:

ANOVA: analysis of variance
ARRIVE: animal research: reporting of *in vivo* experiments
AQP5: aquaporin-5
BSA: bovine serum albumin
CD: cluster of differentiation
ECAD: E-cadherin
hSMG: human submandibular gland
IBA: ionized calcium-binding adaptor molecule 1
NC3Rs: national centre for the replacement, refinement and reduction of animals in research
NIH: National Institutes of Health
NK: natural killer
NHS: National Health Service
SMG: submandibular gland
OCT: optimal cutting temperature
OPCs: oligodendrocyte precursor cells
PBS: phosphate buffered saline
PECAM: platelet endothelial cell adhesion molecule
PFA: paraformaldehyde
UK: United Kingdom
